# Antioxidant polyphenol-rich extracts from the medicinal plants *Antirhea borbonica, Doratoxylon apetalum* and *Gouania mauritiana* protect 3T3-L1 preadipocytes against H_2_O_2_, TNFα and LPS inflammatory mediators by regulating the expression of superoxide dismutase and NF-κB genes

**DOI:** 10.1186/s12950-015-0055-6

**Published:** 2015-02-08

**Authors:** Méry Marimoutou, Fanny Le Sage, Jacqueline Smadja, Christian Lefebvre d’Hellencourt, Marie-Paule Gonthier, Christine Robert-Da Silva

**Affiliations:** UMR Diabète athérothrombose Thérapies Réunion Océan Indien, Inserm U1188 - Université de La Réunion, Plateforme CYROI, 2 rue Maxime Rivière, 97490 Saint-Denis, La Réunion France; Laboratoire de Chimie des Substances Naturelles et des Sciences des Aliments, EA 2212, Université de La Réunion, 15 avenue René Cassin, CS 92003, 97744 Saint-Denis, La Réunion France

**Keywords:** Obesity, Oxidative stress, Inflammation, Antioxidant strategies, Plant polyphenols

## Abstract

**Background:**

Adipose cells responsible for fat storage are the targets of reactive oxygen species (ROS) like H_2_O_2_ and pro-inflammatory agents including TNFα and LPS. Such mediators contribute to oxidative stress and alter inflammatory processes in adipose tissue, leading to insulin resistance during obesity. Thus, the identification of natural compounds such as plant polyphenols able to increase the antioxidant and anti-inflammatory capacity of the body is of high interest. We aimed to evaluate the biological properties of polyphenol-rich extracts from the medicinal plants *A. borbonica*, *D. apetalum* and *G. mauritiana* on preadipocytes exposed to H_2_O_2_, TNFα or LPS mediators.

**Methods:**

Medicinal plant extracts were analysed for their polyphenol contents by Folin-Ciocalteu and UPLC-ESI-MS methods as well as for their free radical-scavenging activities by DPPH and ORAC assays. To assess the ability of polyphenol-rich extracts to protect 3T3-L1 preadipocytes against H_2_O_2_, TNFα or LPS mediators, several parameters including cell viability (MTT and LDH assays), ROS production (DCFH-DA test), IL-6 and MCP-1 secretion (ELISA) were evaluated. Moreover, the expression of superoxide dismutase, catalase and NF-κB genes was explored (RT-QPCR).

**Results:**

All medicinal plants exhibited high levels of polyphenols with free radical-scavenging capacities. Flavonoids such as quercetin, kaempferol, epicatechin and procyanidins, and phenolic acids derived from caffeic acid including chlorogenic acid, were detected. Polyphenol-rich plant extracts did not exert a cytotoxic effect on preadipocytes but protected them against H_2_O_2_ anti-proliferative action. Importantly, they down-regulated ROS production and the secretion of IL-6 and MCP-1 pro-inflammatory markers induced by H_2_O_2_, TNFα and LPS mediators. Such a protective action was associated with an increase in superoxide dismutase antioxidant enzyme gene expression and a decrease in mRNA levels of NF-κB pro-inflammatory transcription factor.

**Conclusion:**

This study highlights that antioxidant strategies based on polyphenols derived from medicinal plants tested could contribute to regulate adipose tissue redox status and immune process, and thus participate to the improvement of obesity-related oxidative stress and inflammation.

## Background

Obesity is characterized by an excessive fat storage in adipose tissue. It contributes to oxidative stress and chronic inflammation which lead to major disorders such as type 2 diabetes and cardiovascular diseases [1-3]. Several mediators causing obesity-related oxidative stress and inflammation have been reported. First, the over-regulated metabolic activity occurring in the adipose tissue during obesity constitutes a main source of reactive oxygen species (ROS) like H_2_O_2_. Continuously generated by the mitochondria, ROS are kept in check by endogenous cellular antioxidant mechanisms such as superoxide dismutase (SOD) and catalase. An imbalance between ROS production and the cellular antioxidant defence system causes oxidative stress [4]. In adipose cells, ROS overproduction alters endoplasmic reticulum and mitochondrial functions as well as cell signalling which induce an inflammatory state [1,5]. The molecular mechanism involved could be partly based on ROS-induced production of the inflammatory cytokine Tumor Necrosis Factor alpha (TNFα). TNFα plays a crucial role in insulin resistance through the down-regulation of insulin-stimulated glucose uptake, insulin receptor auto-phosphorylation and insulin receptor substrate-1 [6]. These effects contribute to the decrease in lipid accumulation within the adipose tissue and to obesity-associated cardiovascular disorders. Thus, additionally to ROS, TNFα represents a second important mediator involved in adipose tissue inflammation during obesity. Its protein levels are significantly increased in adipose tissue of obese humans and its production could result from adipose tissue remodelling characterized by macrophage accumulation [7]. This macrophage infiltration has been reported in adipose tissue of obese patients. It could be responsible for the major part of the locally-produced TNFα, and mainly contributes to the production of the pro-inflammatory cytokine Interleukin-6 (IL-6) through the activation of Nuclear Factor-κB (NF-κB) signalling pathway [5-7]. Much attention has been paid on the mechanisms responsible for macrophage infiltration, and it has been suggested that macrophages present within white adipose tissue could derive from preadipocytes [8,9]. Moreover, adipose cells are able to secrete the chemokine Monocyte Chemoattractant Protein-1 (MCP-1), which is a recruiting factor for circulating monocytes reported to be over-produced in obesity [10]. Similarly to TNFα, both IL-6 and MCP-1 are critically involved in insulin resistance and chronic inflammation [11,12]. More and more studies indicate that such adipokines could also result from the immune response of adipose tissue to an increased level of the lipopolysaccharide (LPS) endotoxin from Gram-negative bacteria during obesity [13]. According to Cani et al. [14], obesity-related excessive dietary lipid intake facilitates the absorption of endotoxins, leading to a higher plasma LPS level. This metabolic endotoxaemia from the gut microbiota could act as a triggering factor in the development of insulin resistance and type 2 diabetes [15-17]. Finally, as ROS and TNFα, LPS may also act as a major obesity-related inflammatory mediator.

No effective pharmacological treatments against obesity-associated oxidative stress and inflammation have been found yet. Thus, the identification of natural compounds able to increase the antioxidant and anti-inflammatory capacity of the body is of high interest. Plant polyphenols constitute the most abundant antioxidants provided by the human diet. More than 5,000 molecules have been identified and classified into main groups, namely phenolic acids, flavonoids, stilbenes, lignans and curcuminoids [18]. A large array of biological properties has been attributed to polyphenols including antioxidant, antibacterial, antiviral and anti-inflammatory activities [19-21]. Recently, we demonstrated that polyphenols protected preadipocytes against mitochondrial alterations and inflammation caused by H_2_O_2_-mediated oxidative stress. Our data also showed that antioxidant and anti-inflammatory effects of polyphenols depended on their chemical nature, dose and time of exposure [22,23]. There is more and more evidence that the consumption of medicinal plants could contribute to increase the daily intake of polyphenols [18]. Three medicinal plants from the Indian Ocean area are traditionally used in order to reduce the incidence of obesity and diabetes, namely *Antirhea borbonica* J.F. Gmelin (Rubiaceae), *Doratoxylon apetalum* (Poir.) Radlk. (Sapindaceae) and *Gouania mauritiana* Lam. subsp. *Mauritiana* (Rhamnaceae). Even if antioxidant and anti-inflammatory properties have been attributed to some medicinal plants from the same species or genus [24-26], there is still a lack of data regarding their effect on adipose cell biology.

Our objective was to evaluate for the first time the antioxidant and anti-inflammatory properties of polyphenol-rich extracts from *A. borbonica*, *D. apetalum* and *G. mauritiana* medicinal plants on preadipocytes exposed to H_2_O_2_, TNFα or LPS. We determined their effects on cell viability, the production of ROS, IL-6 and MCP-1 pro-inflammatory markers, as well as on the expression of genes coding for SOD and catalase antioxidant enzymes, and for NF-κB transcription factor.

## Methods

### Determination of antioxidant polyphenol content in medicinal plant extracts

Plants were selected according to their endemic and indigenous status at Réunion Island based in the Indian Ocean area. All plants tested are commonly used in traditional medicine, although there is a lack of published data concerning their biological effects. Table 1 lists the botanical terms, the voucher number and the parts used. Plant materials were collected during August 2009 and March 2010. They were harvested from various locations in Réunion Island. Botanists of the University of Réunion Island confirmed the identity of all plant materials. After airflow drying (45°C), plant organs were reduced to powder.

**Table 1:** **Global description of the medicinal plants tested**

**Botanical name**	**Family**	**Voucher number**	**Parts used**
*Gouania mauritiana* ^*a*^	Rhamnaceae	RUN-081E	Leaf, stem
Lam. Subsp. mauritiana			
*Antirhea borbonica* ^*b*^	Rubiaceae	RUN-052 F	Leaf, stem
J.F. Gmelin			
*Doratoxylon apetalum* ^*c*^	Sapindaceae	RUN-055E	Leaf
(Poir.) Radlk			

Common names:

^a^: Liane Montbrun.

^b^: Bois d’Osto.

^c^: Bois de gaulette.

### Quantification and identification of polyphenols in medicinal plant extracts

Polyphenol-rich extracts from medicinal plants were obtained after dissolving each plant powder (2 g) in 20 mL of an aqueous acetonic solution (70%, v/v). After incubation at 4°C for 90 min, the mixture was centrifuged at 3500 rpm at 4°C for 20 min and polyphenol-rich supernatants were collected and stored at −80°C until analysis. To determine polyphenol contents in plant extracts, Folin-Ciocalteu test was used [27]. Briefly, 25 μL plant extract, 125 μL Folin-Ciocalteu’s phenol reagent (Sigma-Aldrich, Germany) and 100 μL sodium carbonate were added in a 96-well microplate and incubated at 54°C for 5 min and then at 4°C for 5 min. The absorbance was measured at 765 nm (FLUOstar Optima, Bmg Labtech, Germany). A calibration curve was prepared using a standard solution of gallic acid (Sigma-Aldrich, Germany). Total phenol content was expressed as g gallic acid equivalent (GAE) / 100 g plant powder. Total flavonoids were also measured using a colorimetric assay adapted from Zhishen et al. [28]. For this experiment, 100 μL of each polyphenol-rich plant extract were placed in a 96-well microplate with 6 μL of 5% aqueous NaNO_2_. After 5 min, 6 μL of 10% aqueous AlCl_3_ were added and the mixture was vortexed. Then, after 1 min, 40 μL of 1 M NaOH were added. The absorbance was measured at 510 nm (FLUOstar Optima, Bmg Labtech, Germany). A calibration curve was prepared using a standard solution of catechin (Sigma-Aldrich, Germany). Total flavonoid contents were expressed as g catechin equivalent (CE) / 100 g plant powder. The identification of polyphenols was achieved by an UPLC-ESI-MS analysis (Agilent Technologies, USA) according to the method previously described by Mossalayi et al. with slight modifications [29].

### Free radical-scavenging activity of polyphenol-rich extracts from medicinal plants

#### Oxygen radical absorbance capacity (ORAC) assay

ORAC is based on the decrease of fluorescein fluorescence in presence of the chemical oxidant 2,2′-azobis [2-methyl-propionamidin] dihydrochloride (AAPH). The assay was done as described by Huang et al. with some modifications [30]. Briefly, 25 μL sample were placed in a 96-well black microplate, and 150 μL of 8 × 10^−5^ mM fluorescein (Sigma-Aldrich, Germany) were added. After 15 min at 37°C, 25 μL of 153 mM AAPH (Sigma-Aldrich, Germany) were added to each well. Then, the fluorescence was measured for 1 h 40 min at a wavelength of excitation and emission of 485 nm and 530 nm, respectively (Infinite 200, Tecan, USA). The results were based on the area under the curve of fluorescence decay over time and compared with a Trolox (Sigma-Aldrich, Germany) calibration curve. Free radical-scavenging activities of polyphenol-rich plant extracts were expressed as mM Trolox equivalent.

#### 2,2-Diphenyl-1-picrylhydrazyl (DPPH) assay

The scavenging activity on DPPH radical was measured according to the method described by Yang et al. with slight modifications [31]. Briefly, 0.25 mM DPPH (Sigma-Aldrich, Germany) diluted in methanol was incubated with each polyphenol-rich plant extract or 100 μM antioxidant standard (vitamin C, the flavonoids catechin or quercetin, and the phenolic acids gallic, caffeic, ferulic or chlorogenic acids, Sigma-Aldrich, Germany). After 25 min at 25°C, the optical density (OD) was read at 517 nm (FLUOstar Optima, Bmg Labtech, Germany). The percentage of free radical-quenching activity of DPPH was determined according to the following formula:

$$ \mathrm{Antioxidant}\ \mathrm{capacity}\ \left(\%\right) = \left[\left(\mathrm{O}\mathrm{D}\ \mathrm{control}\ \hbox{--}\ \mathrm{O}\mathrm{D}\ \mathrm{sample}\right)\ /\ \mathrm{O}\mathrm{D}\ \mathrm{control}\right] \times 100 $$

### Evaluation of the effect of polyphenol-rich plant extracts on preadipocyte viability

Mouse preadipocytes obtained from ATCC CL-173 were cultivated in Dulbecco’s Modified Eagle’s Medium containing 25 mM glucose, 10% heat-inactivated fetal bovine serum, L-glutamin (5 mM), streptomycin (2 μg/mL) and penicillin (50 μU/mL). The cell culture condition was in a humidified 5% CO_2_ incubator at 37°C.

#### MTT assay

Mitochondrial metabolic activity of cells was determined by 3-(4,5-dimethyl-thiazol-2-yl)-2,5-diphenenyl tetrazolium bromide (MTT) reduction assay. 3T3-L1 cells were seeded overnight in 96-well plates at a density of 6 × 10^3^ cells per well. The day after, the medium was removed and cells were treated with each polyphenol-rich plant extract (0–200 μM GAE) for 24, 48, and 72 h. Five hours before the end of the incubation, 20 μL MTT dye (5 mg/mL, Sigma-Aldrich, Germany) were added into each well to allow the formation of the dark blue formazan crystals generated by living cells. Then, the medium was removed and 100 μL of DMSO were added to dissolve the crystals. Absorbance was read at 595 nm (FLUOstar Optima, Bmg Labtech, Germany).

#### Trypan blue exclusion method

3T3-L1 cells were seeded overnight in 12-well plates at a density of 50 × 10^3^ cells per well. The day after, the medium was removed and cells were treated with each polyphenol-rich plant extract (25 μM GAE) for 24 and 48 h. Then, cells were detached with trypsin, collected in the medium and centrifuged (2000 rpm, 24°C, 4 min). Finally, preadipocytes were counted using Trypan Blue solution (Sigma-Aldrich, Germany).

#### Lactate dehydrogenase (LDH) release assay

LDH activity was measured using a commercial cytotoxicity assay kit (Sigma-Aldrich, Germany). For this test, 3T3-L1 cells were treated with each polyphenol-rich plant extract (25 μM GAE) for 24 and 48 h. According to the manufacturer’s instructions, 50 μL of cell culture medium were added to 100 μL of LDH reagent in 96-well plates, then the mixture was incubated at room temperature during 30 min. The reaction was stopped by adding 15 μL of 1 M HCl. The absorbance was read at 490 nm (FLUOstar Optima, Bmg Labtech, Germany).

### Evaluation of the effect of polyphenol-rich plant extracts on ROS production from preadipocytes

The level of intracellular ROS was assessed by measuring the oxidation of DCFH-DA, according to the method previously published [22,23]. DCFH-DA diffuses through the cell membrane and is deacetylated by cellular esterases to the non-fluorescent DCFH. Intracellular ROS are able to oxidize DCFH to the fluorescent 2,7-dichlorofluorescein (DCF), whose intensity of fluorescence is directly proportional to the levels of intracellular ROS. Briefly, cells were cultured in 96-well black microplates (6 × 10^3^ cells/well) for 24 h. Then, the medium was removed and replaced by PBS containing 10 μM of DCFH-DA (Sigma-Aldrich, Germany) and cells were kept in a humidified atmosphere (5% CO_2_, 37°C) for 45 min. Next, cells were exposed to each polyphenol-rich plant extract (25 μM GAE) or caffeic acid standard as a positive control (25 μM), or treated with H_2_O_2_ (200 μM, Sigma-Aldrich, Germany), TNFα (5 ng/mL, eBioscience, UK) or LPS from *E. coli* K-235 (1 μg/mL, Sigma-Aldrich, Germany) in the presence or not of each plant extract (25 μM GAE) or caffeic acid standard (25 μM). After 1 h, fluorescence was measured at an excitation wavelength of 492 nm and an emission wavelength of 520 nm (FLUOstar Optima, Bmg Labtech, Germany).

### Evaluation of the effect of polyphenol-rich plant extracts on the production of pro-inflammatory cytokines from preadipocytes

Cells were pre-incubated overnight in 24-well plates at a density of 37 × 10^3^ cells/well. The day after, they were treated with H_2_O_2_ (200 μM), TNFα (5 ng/mL) or LPS (1 μg/mL) in the presence or not of each polyphenol-rich plant extract (25 μM GAE). After 24 h, cell culture media were collected and stored at −20°C until analysis. Levels of the pro-inflammatory markers IL-6, TNFα and MCP-1 were determined by using specific ELISA kits (eBioscience, UK) and normalized according to total cellular protein amounts determined by Bradford assay [32].

### Evaluation of the effect of polyphenol-rich plant extracts on the expression of SOD, catalase and *NF-κB* genes from preadipocytes

Cells were pre-incubated overnight in 6-well plates at a density of 150 × 10^3^ cells/well. The day after, they were treated with H_2_O_2_ (200 μM), TNFα (5 ng/mL) or LPS (1 μg/mL) in the presence or not of each polyphenol-rich plant extract (25 μM GAE). After 24 h, total RNA was isolated with TRIzol™ (Invitrogen, France). An amount of 2 μg of total RNA were reverse-transcribed (RT) using Random hexamer primers (Eurogentec, Belgium) with Superscript™ II (Invitrogen, France). As previously described by Awada et al. [33], the quantitative polymerase chain reaction (QPCR) was conducted using SYBR green™ master Mix (Eurogentec, Belgium). Primer and probe sequences are listed on Table 2. Results were analyzed using 7500 system SDS software (Applied Biosystems, France) and the relative expression of SOD, catalase and p50 NF-κB genes was normalized against the expression level of glyceraldehyde-3-phosphate dehydrogenase (GAPDH) gene.

**Table 2:** **Primers used for RT-QPCR analysis**

**Mouse gene**	**Forward primer**	**Reverse primer**	**Detection**
**NF-κB**	CATTTGAACACTGCTTTGACTCACT	GTGATGGGCCTTCACACACA	Sybrgreen
**Cu/Zn SOD**	GCAGGGAACCATCCACTT	TACAACCTCTGGAACCGT	Sybrgreen
**Catalase**	CCTCCTCGTTCAGGATGTGGTT	CGAGGGTCACGAACTGTGTCAG	Sybrgreen
**GAPDH**	TTCACCACCATGGAGAAGGC	GGCATGGACTGTGGTCATGA	Sybrgreen

### Statistical analysis

Data were expressed as means ± SEM from three independent experiments (three different passages), themselves based on triplicates. Statistical analysis was performed by using one-way ANOVA followed by the Tukey’s multiple comparisons test. Significant differences were considered for p value < 0.05 (PRISM software, USA).

## Results

### Polyphenol content and antioxidant activities of medicinal plant extracts

**Figure 1:**
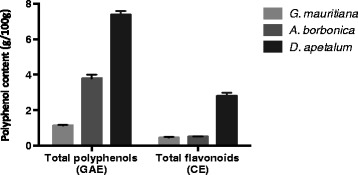
**Total polyphenol contents of** ***A. borbonica, D. apetalum and G. mauritiana*** **plant extracts.** Polyphenols and flavonoids levels were determined by using colorimetric assays and respectively expressed as g gallic acid equivalent (GAE) / 100 g plant powder or g catechin equivalent (CE) / 100 g plant powder. Data are means ± SEM of three independent experiments.

**Figure 2:**
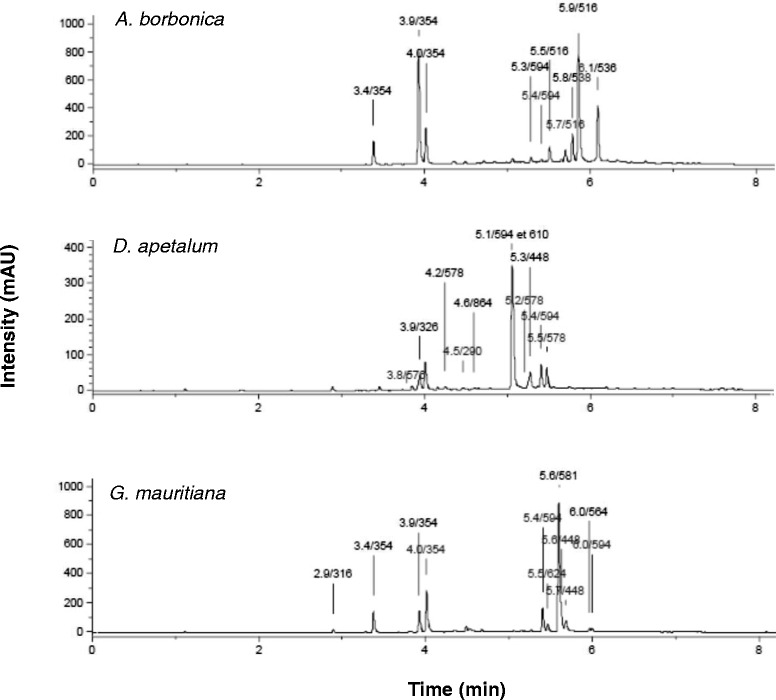
**Identification of polyphenols from** ***A. borbonica, D. apetalum and G. mauritiana*** **plant extracts.** Polyphenol-rich plant extracts were analysed by UPLC-ESI-MS method (320 nm). Compounds were identified according to their retention time (min)/molecular weight (Da). Polyphenols detected from *A. borbonica* plant extract were chlorogenic acids (3.4/354; 3.9/354; 4.0/354), kaempferol-O-hexoside-O-rhamnoside (5.3/594; 5.4/594) and dicaffeoylquinic acid (5.5/516; 5.7/516, 5.9/516). Polyphenols detected from *D. apetalum* plant extract were procyanidins including dimer type A (3.8/576), dimer type B (4.2/578; 5.2/578) and trimer type A (4.6/864), coumaric acid-O-hexoside (3.9/326), epicatechin (4.5/290), kaempferol-O-hexoside-O-rhamnoside (5.1/594; 5.4/594), quercetin-O-rutinoside (5.1/610) and kaempferol 3-O-hexoside (5.3/448). Polyphenols detected from *G. mauritiana* plant extract were protocatechuic acid-O-hexoside (2.9/316), chlorogenic acids (3.4/354; 3.9/354; 4.0/354), kaempferol-O-hexoside-O-rhamnoside (5.4/594), isorhamnetin-O-hexoside-O-rhamnoside (5.5/624), quercetin-O-xyloside-O-rhamnoside (5.6/581), kaempferol-O-hexoside (5.6/448), quercetin-O-rhamnoside (5.7/448), kaempferol-O-rhamnoside-O-xyloside (6.0/564) and isorhamnetin-O-rhamnoside-O-xyloside (6.0/594).

Total polyphenol content from medicinal plant acetonic extracts was evaluated by using Folin-Ciocalteu assay. As shown on Figure 1, *D. apetalum* extract exhibited the highest polyphenol content (7.0% GAE, w/w) followed by *A. borbonica* (3.8% GAE, w/w) and *G. mauritiana* extracts (1.0% GAE, w/w). As flavonoids are the most abundant polyphenols in the human diet and may account for about two thirds of the total intake [18,34], total flavonoid content from plant extracts was determined. Data reported on Figure 1 demonstrated the presence of flavonoids which were also more abundant in *D. apetalum* extract (2.8% CE, w/w) than in both *A. borbonica* and *G. mauritiana* extracts (0.49 and 0.44% CE, w/w, respectively). An UPLC-ESI-MS analysis of plant polyphenols led to identify several phenolic acids and flavonoids commonly found in plants including caffeic acid, chlorogenic acid, coumaric acid, protocatechuic acid, quercetin, kaempferol and catechin derivatives. (Figure 2). Interestingly, *D. apetalum* extract identified as the most abundant source of polyphenols was characterized by the presence of procyanidins under dimer (type A and type B) and trimer (type A) forms.

**Figure 3:**
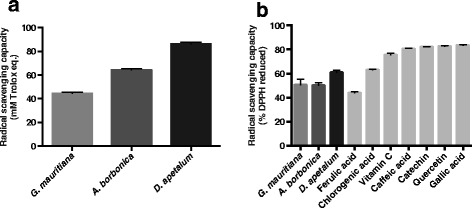
**Antioxidant activities of polyphenol-rich plant extracts. (a)** Free radical-scavenging capacity of plant extracts was assessed by ORAC assay and expressed as mM Trolox equivalent. **(b)** Free radical-scavenging activities of plant extracts and standard polyphenols and vitamin C (100 μM) were measured through DPPH method and expressed as % DPPH reduced. Data are means ± SEM of three independent experiments.

To assess if the presence of polyphenols in medicinal plant extracts provided them an antioxidant capacity, both ORAC and DPPH assays were used. Results from ORAC method showed that all polyphenol-rich extracts exhibited a strong antioxidant capacity ranging from 44–86 mM Trolox equivalent, with *D. apetalum* extract identified as the most abundant source of antioxidant polyphenols (Figure 3a). Data from DPPH test confirmed that all plant polyphenol-rich extracts exerted a free radical-scavenging activity reaching 50.2 ± 2.0% for *A. borbonica* extract, 50.7 ± 4.6% for *G. mauritiana* extract and 61.1 ± 1.6% for *D. apetalum* extract (Figure 3b). Such an antioxidant capacity was close to that measured for ferulic acid or chlorogenic acid standards (44.0 ± 0.6 and 63.2 ± 0.2%, respectively) whereas it was lower than that of other antioxidant standards such as vitamin C, caffeic acid, catechin, quercetin and gallic acid ranging from 75.6 ± 1.1 to 83.6 ± 0.1%. Accordingly, data obtained above from UPLC-ESI-MS analysis led to demonstrate the presence of both ferulic and chlorogenic acids in plant extracts.

### Impact of polyphenol-rich plant extracts on preadipocyte viability

**Figure 4:**
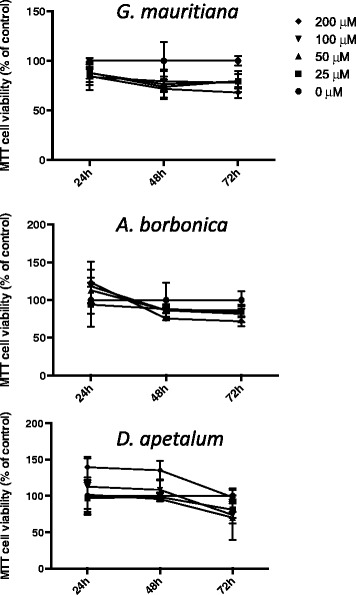
**Effect of polyphenol-rich plant extracts on preadipocyte mitochondrial metabolic activity.** Cells were exposed to each plant extract (0–200 μM GAE) for 24, 48 and 72 h. Then, the mitochondrial metabolic activity of cells was measured by MTT assay. Data are means ± SEM of three independent experiments.

**Figure 5:**
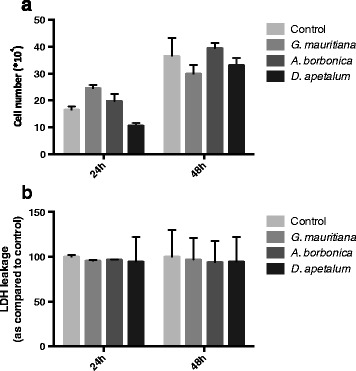
**Effect of polyphenol-rich plant extracts on preadipocyte viability.** Cells were exposed to each plant extract (25 μM GAE) for 24 and 48 h. **(a)** In order to assess cell growth, cells were counted manually by using Trypan blue reagent. **(b)** To evaluate cell death, LDH activity in the cell culture medium was determined by using an enzymatic kit. Data are means ± SEM of three independent experiments.

In order to evaluate the potential cytotoxic action of polyphenol-rich plant extracts, 3T3-L1 preadipocyte viability was evaluated through MTT assay measuring the mitochondrial reductase activity of metabolizing cells. An analysis of dose-dependent effects (0–200 μM GAE) during 24, 48 and 72 h was performed. At the dose tested, no statistical difference was observed between the control and cells treated with each plant extract (Figure 4). MTT assay reflected the mitochondrial activity, thus it was important to determine the number of living cells. As illustrated here for the dose of 25 μM GAE, there was also no statistically significant effect of polyphenol-rich extracts on the cell growth assessed by cell counting (Figure 5a), or on the cell death explored by LDH test (Figure 5b). Taken together, these results indicate that polyphenol-rich extracts from medicinal plants did not exert a cytotoxic effect on preadipocytes.

### Impact of polyphenol-rich plant extracts on preadipocytes exposed to H_2_O_2_ mediator

**Figure 6:**
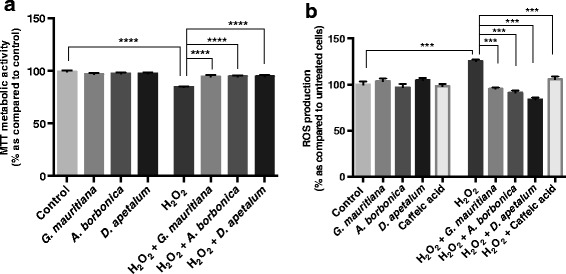
**Effect of polyphenol-rich plant extracts on the viability and ROS production of preadipocytes exposed to H**_**2**_ **O**_**2**_ **. (a)** Cells were exposed to each plant extract (25 μM GAE) or treated with H_2_O_2_ (200 μM) in the presence or not of each plant extract (25 μM GAE) for 24 h. Then, the mitochondrial metabolic activity of cells was measured by MTT assay. **(b)** Cells were exposed to 10 μM of DCFH-DA for 45 min at 37°C and then were treated with each plant extract (25 μM GAE) or caffeic acid as a positive control (25 μM), or treated with H_2_O_2_ (200 μM) in the presence or not of each plant extract (25 μM GAE) or caffeic acid (25 μM) for 1 h. Data are means ± SEM of three independent experiments. ***: p < 0.001, ****: p < 0.0001 as compared to H_2_O_2_.

To explore the effect of polyphenol-rich plant extracts against H_2_O_2_-induced oxidative stress, preadipocytes were exposed to H_2_O_2_ (200 μM) in the presence or absence of each polyphenol-rich plant extract (25 μM GAE) during 24 h. The choice of H_2_O_2_ and polyphenol doses was based on previous published works [22,23,35]. As shown on Figure 6a, MTT assay demonstrated that H_2_O_2_ decreased the mitochondrial metabolic activity of cells (from 100 to 84% as compared to untreated cells, p < 0.0001). Interestingly, all polyphenol-rich extracts from medicinal plants were able to reverse H_2_O_2_ anti-proliferative action. This result could be explained by their strong antioxidant activity shown on Figure 3 and raised the question about the impact of polyphenol-rich plant extracts on ROS production from preadipocytes exposed to H_2_O_2_.

When ROS production is not compensated by cellular antioxidant defence system, oxidative stress occurs. In order to investigate the potential protective action of polyphenol-rich plant extracts against oxidative stress, we measured ROS production after co-treating preadipocytes with H_2_O_2_ (200 μM) and each plant extract (25 μM GAE) during 1 h. In accordance with our previous studies [22,23] and as described above for DPPH method, caffeic acid was used as a conventional antioxidant for a positive control. Whereas plant extracts and caffeic acid did not modulate ROS basal production as compared to untreated cells, H_2_O_2_ induced a significant increase in ROS generation (from 100 to 125%, p < 0.001). All polyphenol-rich plant extracts significantly reduced H_2_O_2_-induced ROS production similarly to caffeic acid (Figure 6b).

**Figure 7:**
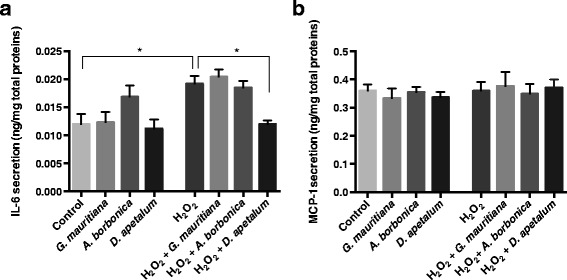
**Effect of polyphenol-rich plant extracts on IL-6 and MCP-1 secretion from preadipocytes exposed to H**_**2**_ **O**_**2**_ **.** Cells were exposed to each plant extract (25 μM GAE) or treated with H_2_O_2_ (200 μM) in the presence or not of each plant extract (25 μM GAE) for 24 h. Then, levels of IL-6 **(a)** and MCP-1 **(b)** were measured by ELISA kits. Data are means ± SEM of three independent experiments. *: p < 0.05 as compared to H_2_O_2_.

To assess if oxidative stress induced by H_2_O_2_ affects preadipocyte inflammatory response, it was relevant to measure the production of key adipokines such as TNFα, IL-6 and MCP-1 from cells exposed to H_2_O_2_. In agreement with our previous data [23], TNFα was not detectable in any condition in our study (data not shown). Concerning IL-6, the basal level detected was 0.12 ± 0.02 ng/mg proteins and was not affected by polyphenol-rich extracts (Figure 7a). When cells were exposed to H_2_O_2_ for 24 h, IL-6 level significantly increased to 0.20 ± 0.01 ng/mg proteins (p < 0.05). In co-exposition condition, only polyphenol-rich extract from *D. apetalum* plant reversed this up-regulation of IL-6 production, suggesting an anti-inflammatory activity which may result from the activity of some specific compounds such as procyanidins shown on Figure 2. Regarding MCP-1 secretion, no statistically significant modulation was observed in cells co-treated or not with H_2_O_2_ and polyphenol-rich plant extracts (Figure 7b). This result suggests that H_2_O_2_-induced oxidative stress may specifically alter the secretion of some pro-inflammatory adipokines depending on their nature.

### Impact of polyphenol-rich plant extracts on preadipocytes exposed to TNFα mediator

**Figure 8:**
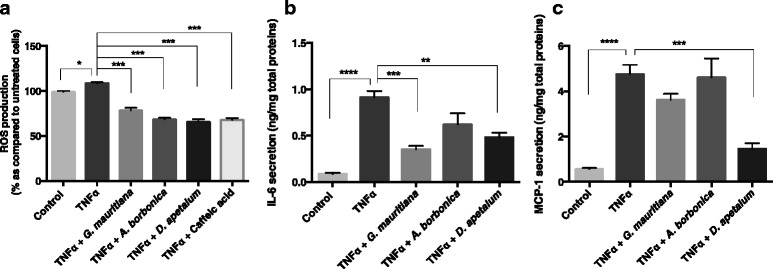
**Effect of polyphenol-rich plant extracts on ROS, IL-6 and MCP-1 production from preadipocytes exposed to TNFα. (a)** Cells were exposed to 10 μM of DCFH-DA for 45 min at 37°C and then were treated with TNFα (5 ng/mL) in the presence or not of each plant extract (25 μM GAE) or caffeic acid (25 μM) for 1 h. **(b)** Cells were treated with TNFα (5 ng/mL) in the presence or not of each plant extract (25 μM GAE) for 24 h. Then, levels of IL-6 were measured by ELISA kit. **(c)** According to the experimental condition used for IL-6 detection, levels of MCP-1 were measured by ELISA kit. Data are means ± SEM of three independent experiments. *: p < 0.05, **: p < 0.01, ***: p < 0.001, ****: p < 0.0001 as compared to TNFα.

TNFα has been reported to play an important role in adipose tissue inflammation during obesity. However, its effect on preadipocytes has been poorly studied. Here, to explore the ability of medicinal plant extracts to modulate the potential action of TNFα on ROS production, 3T3-L1 cells were exposed to the pro-inflammatory mediator (5 ng/mL) in the presence or not of polyphenol-rich plant extracts (25 μM GAE) or caffeic acid standard. TNFα significantly increased ROS levels (from 100 to 109%, p < 0.05) (Figure 8a). Interestingly, all polyphenol-rich plant extracts counteracted TNFα-induced oxidative stress at the same extent than that of caffeic acid positive control. Levels of IL-6 and MCP-1 in cell culture media were also determined. As mentioned above for cells exposed to H_2_O_2_, IL-6 production was significantly affected. Indeed, TNFα induced a 10-fold increase in IL-6 basal level detected at 0.08 ± 0.01 ng/mg proteins (Figure 8b). This pro-inflammatory effect of TNFα was significantly reduced in cells co-exposed to polyphenol-rich extracts from *G. mauritiana* and *D. apetalum* extracts. It appeared that *G. mauritiana* extract exhibited the highest anti-inflammatory action by reversing IL-6 level from 0.91 ± 0.07 to 0.35 ± 0.04 ng/mg proteins, as compared to *D. apetalum* extract (from 0.91 ± 0.07 to 0.48 ± 0.04 ng/mg proteins, respectively). Interestingly, as *G. mauritiana* extract was not able to modulate H_2_O_2_-induced IL-6 elevation reported above, our results suggest that the anti-inflammatory effect of a plant extract may also depend on the nature of the inflammatory mediator. Moreover, in contrast to H_2_O_2_ mediator, TNFα significantly increased MCP-1 basal production (from 0.56 ± 0.05 to 4.75 ± 0.4 ng/mg proteins, p < 0.0001) (Figure 8c). Only polyphenol-rich extract from *D. apetalum* plant reversed this up-regulation of MCP-1 secretion. This is in agreement with the results described previously showing a specific anti-inflammatory property of *D. apetalum* extract against H_2_O_2_-induced IL-6 elevation.

### Impact of polyphenol-rich plant extracts on preadipocytes exposed to LPS mediator

**Figure 9:**
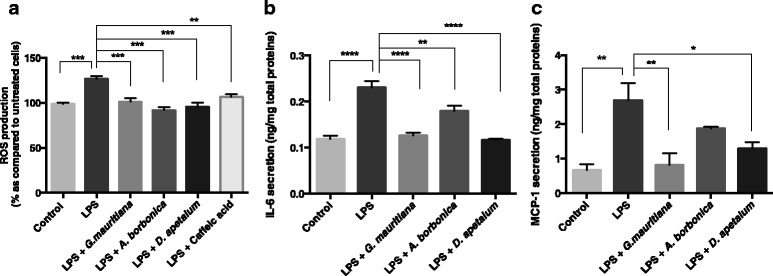
**Effect of polyphenol-rich plant extracts on ROS, IL-6 and MCP-1 production from preadipocytes exposed to LPS. (a)** Cells were exposed to 10 μM of DCFH-DA for 45 min at 37°C and then were treated with *E. coli* LPS (1 μg/mL) in the presence or not of each plant extract (25 μM GAE) or caffeic acid (25 μM) for 1 h. **(b)** Cells were treated with *E. coli* LPS (1 μg/mL) in the presence or not of each plant extract (25 μM GAE) for 24 h. Then, levels of IL-6 were measured by ELISA kit. **(c)** According to the experimental condition used for IL-6 detection, levels of MCP-1 were measured by ELISA kit. Data are means ± SEM of three independent experiments. *: p < 0.05, **: p < 0.01, ***: p < 0.001, ****: p < 0.0001 as compared to LPS.

LPS bacterial endotoxin has been reported to contribute to adipose tissue inflammation and insulin resistance, however the mechanism still remains unclear. To explore the potential effect of polyphenol-rich plant extracts against LPS action on preadipocytes, cells were treated with the bacterial mediator (1 μg/mL) in the presence or not of each plant extract (25 μM GAE). Whereas LPS significantly elevated ROS production (from 100 to 126%, p < 0.001), each polyphenol-rich plant extract counteracted this pro-oxidant damage as caffeic acid positive control (Figure 9a). As shown on Figure 9b, LPS also induced a 2-fold increase in IL-6 basal level (p < 0.0001). All polyphenol-rich plant extracts were able to protect against LPS pro-inflammatory effect. Both *D. apetalum* and *G. mauritiana* extracts exerted the highest anti-inflammatory activity (p < 0.0001). In contrast to *A. borbonica* extract which partially protected cells, *D. apetalum* and *G. mauritiana* extracts totally reversed the effect of LPS by reducing IL-6 level from 0.23 ± 0.05 to 0.10 ± 0.01 and 0.12 ± 0.07 ng/mg proteins, respectively. Regarding MCP-1, whereas LPS induced a 3-fold increase in the basal production detected at 2.68 ± 0.5 ng/mg proteins (p < 0.01), both *G. mauritiana* and *D. apetalum* extracts exhibited a significant anti-inflammatory action by decreasing LPS-induced level to 0.81 ± 0.34 and 1.29 ± 0.18 ng/mg proteins, respectively (Figure 9c). As observed previously for H_2_O_2_ and TNFα mediators, such a specific protective effect of some plant extracts against LPS-induced inflammation highlights that the anti-inflammatory action of plant extracts may depend on their polyphenolic content, the nature of the inflammatory mediator involved as well as the nature of the cytokine/chemokine affected.

### Impact of polyphenol-rich plant extracts on the expression of SOD, catalase and NF-κB genes from preadipocytes exposed to H_2_O_2_, TNFα or LPS mediators

**Figure 10:**
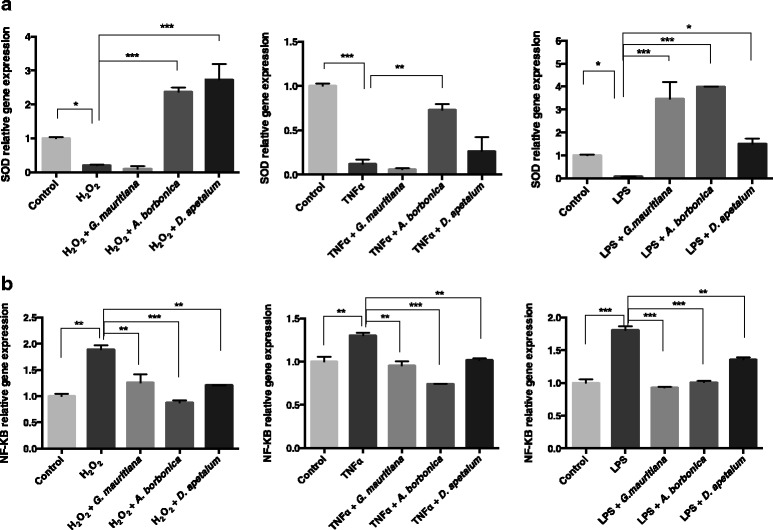
**Effect of polyphenol-rich plant extracts on the expression of SOD and NF-κB genes from preadipocytes exposed to H**_**2**_ **O**_**2,**_ **TNFα or LPS.** Cells were treated with H_2_O_2_ (200 μM), TNFα (5 ng/mL) or *E. coli* LPS (1 μg/mL) in the presence or not of each plant extract (25 μM GAE) for 24 h. Then, the relative expression of SOD **(a)** and NF-κB **(b)** genes was measured by RT-QPCR and normalized against the expression level of GAPDH gene. Data are means ± SEM of three independent experiments. *: p < 0.05, **: p < 0.01, ***: p < 0.001 as compared to H_2_O_2_, TNFα or LPS.

To understand the antioxidant and anti-inflammatory properties of polyphenol-rich plant extracts, their effects on the expression of genes coding for SOD and catalase antioxidant enzymes, as well as for NF-κB pro-inflammatory transcription factor were explored. Whereas catalase gene expression was not affected (data not shown), SOD mRNA levels were significantly decreased on cells exposed to H_2_O_2_, TNFα or LPS mediators (Figure 10a). This result agrees with data described above showing the ability of such mediators to induce ROS production. Interestingly, polyphenol-rich extracts were able to reverse the effect by up-regulating SOD gene expression, depending on the plant and the mediator considered. Moreover, H_2_O_2_, TNFα and LPS mediators induced a significant elevation of NF-κB gene expression which was remarkably counteracted by all polyphenol-rich plant extracts (Figure 10b).

## Discussion

Few studies have focused on the redox status and inflammatory response of preadipocytes during obesity despite the fact that preadipose cells play an important role by governing the development of adipose tissue and fat mass. Some dietary polyphenols such as phenolic acids have been shown to affect preadipocyte viability by inducing apoptosis through a Fas- and mitochondrial-mediated pathway [36]. Moreover, our recent data demonstrated that the ability of polyphenols to modulate preadipocyte growth depended on their chemical nature and concentration [23]. Thus, it was important to evaluate polyphenol levels in medicinal plant extracts in order to assess a potential cytotoxic effect depending on the dose used. It appeared that all plant extracts exhibited a high polyphenol content ranging from 1-7% GAE (w/w) with *D. apetalum* extract identified as the most abundant source. Major flavonoids like quercetin and catechin as well as phenolic acids derived from caffeic acid such as chlorogenic acids, known to be abundant in foods and medicinal plants [18,37], were identified. Similar polyphenols have been reported for cocoa seed (6-8%, w/w) as well as for green coffee (3%, w/w) [18,38]. Results from Hsu et al. [39] demonstrated that such polyphenols caused a cell cycle arrest of 3T3-L1 preadipocytes in the G_1_ phase. Here, none of the polyphenol-rich plant extract affected preadipocyte viability measured by MTT and LDH assays, suggesting the absence of cytotoxic effect.

In order to explore the impact of polyphenol-rich plant extracts against oxidative stress, preadipocytes were co-exposed to H_2_O_2_. All polyphenol-rich extracts were able to reverse H_2_O_2_-mediated anti-proliferative action and ROS production. This protective effect could be attributed to the presence of high levels of polyphenols exhibiting a strong free radical-scavenging activity as demonstrated by using both DPPH and ORAC methods. Such antioxidant properties of polyphenols have been largely reported and could be explained not only by the scavenging of free radicals, but also by the modulation of the function of mitochondria which constitutes the major cellular source of ROS [4]. Indeed, several polyphenols including the family of phenolic acids detected here in medicinal plant extracts have been reported to affect the activity of mitochondrial components mainly involved in the regulation of free radical generation, by increasing levels of SOD, glutathione, glutathione peroxidase and glutathione S-transferase [40]. In the present study, the elevation of ROS production induced by H_2_O_2_ was accompanied by a significant reduction of SOD gene expression which was counteracted by both *A. borbonica* and *D. apetalum* extracts. H_2_O_2_ also induced an increase in the production of IL-6 pro-inflammatory cytokine which was down-regulated only by the polyphenol-rich extract from *D. apetalum* plant. Such a pro-inflammatory action of H_2_O_2_ was associated with an elevation of the expression of NF-κB, established as a key transcription factor involved in the regulation of IL-6 secretion in several cell types [41-44]. Interestingly, all polyphenol-rich plant extracts significantly down-regulated NF-κB gene expression induced by H_2_O_2_. Concerning the inhibitory effect of *D. apetalum* extract on IL-6 secretion, it may be mediated by the presence of specific compounds. Our work led to identify polyphenols such as chlorogenic acid at a very high level in *D. apetalum* extract. The ability of such plant polyphenols to down-regulate NF-κB gene expression may partly explain their effect on the decrease in pro-inflammatory cytokine production. However, other mechanisms including the regulation of NF-κB protein translocation or Inhibitor-κB (IκB) phosphorylation cannot be excluded. Interestingly, several literature data have reported that chlorogenic acid is very effective against inflammation due to its ability to decrease IκB phosphorylation as well as the nuclear translocation of NF-κB [45-47]. It is also noteworthy that *D. apetalum* extract exhibited a 6-fold higher level of flavonoids as compared to other plant extracts. Such compounds could also contribute to explain *D. apetalum* extract anti-inflammatory action. For illustration, quercetin which is one of the most antioxidant flavonoids commonly found in plants, has been reported to strongly down-regulate H_2_O_2_-induced IL-6 secretion [23,48].

Importantly, the present work highlights that H_2_O_2_ as well as TNFα and LPS provoked oxidative and pro-inflammatory damages on preadipose cells. Noticeably, the inflammatory response of preadipocytes exposed to these major obesity-related inflammatory mediators depended on the nature of the mediator considered. Indeed, whereas H_2_O_2_-induced inflammation was characterized by an increase in IL-6 secretion without any modification of MCP-1 level, TNFα and LPS up-regulated the production of both IL-6 and MCP-1 pro-inflammatory markers. This result is in agreement with literature data reporting the ability of TNFα and LPS to change the expression and secretion of several adipokines. Indeed, it was nicely described that 34 genes including those encoding for IL-6 and MCP-1 were induced in a dose dependent manner in TNFα-treated human and mouse adipocytes. Such pro-inflammatory effects were also reported to be time-dependent [40,49]. Regarding LPS action, Chirumbolo et al. [50] recently demonstrated that IL-6 secretion was constantly up-regulated by prolonging incubation with LPS for 24 h, whereas the production of other inflammatory markers such as the chemokine Macrophage Inflammatory Protein-1α (MIP-1α) was induced within the first 2–8 h. Mechanistically, it was established that the expression of these cytokines was regulated predominantly by NF-κB protein [40,49,50]. Accordingly, our present work provided evidence that TNFα and LPS induced a significant increase in NF-κB gene expression, similarly to H_2_O_2_. Such TNFα or LPS pro-inflammatory response pattern associated with their oxidative damages through ROS generation may play a role in the regulation of the immune phenotype of preadipose cells, and affect their normal differentiation. Indeed, ROS, IL-6 and TNFα were reported to impair the normal differentiation of preadipocytes and lipid accumulation contributing to inflammation-induced adipose tissue dysregulation and insulin resistance [1,5]. Our study also showed that TNFα and LPS significantly increased the secretion of MCP-1, which is known as a major chemokine involved in macrophage infiltration into the adipose tissue. Here, taking into account the preadipocyte inflammatory state in response to major mediators such as H_2_O_2_, TNFα or LPS, our data contributed to support the demonstration of the crucial impact of inflammation on preadipocyte capacity to proliferate/differentiate as well as on their ability to recruit other cells such as macrophages. Several literature data have established a link between adipose tissue and immuno-competent cells. This link is illustrated by the great cellular plasticity exhibited by preadipocytes to be very efficiently and rapidly converted into macrophages in an inflammatory environment. This preadipocyte phenotype conversion disappears when preadipose cells stop proliferating and differentiate into adipocytes. Thus, such an ability of preadipocytes to function as macrophage-like cells may play a crucial role in the involvement of adipose tissue in inflammatory processes [8,9,51]. Moreover, through the preadipocyte secretion of MCP-1 which participates to the recruitment of macrophages, immune processes in adipose tissue may be reinforced in response to TNFα and LPS mediators [10].

Our data provided the first evidence that polyphenol-rich extracts from *A. borbonica*, *D. apetalum* and *G. mauritiana* plants inhibited ROS production and the down-regulation of SOD gene expression mediated by H_2_O_2_, TNFα and LPS mediators. In agreement with literature data, the absence of effect of such mediators on catalase gene expression may reflect a specific action on ROS level regulation through SOD enzyme modulation [40]. This antioxidant effect was also associated with a decrease in preadipocyte pro-inflammatory state through the reduction of IL-6 and MCP-1 secretion as well as NF-κB gene expression. Thus, such an anti-inflammatory action of antioxidant polyphenols from medicinal plants emphasizes their potential capacity to modulate the link between adipose tissue and immune processes during obesity, by regulating key signalling pathways involving SOD and NF-κB proteins. There is a growing literature data regarding the evaluation of anti-inflammatory therapeutic strategies based on phenolic acids like caffeic acid derivatives or flavonoids such as those present in green tea, blueberry, grape seed and several other plants derived from traditional medicine [52-56]. Interestingly, our results showed that polyphenol-rich plant extracts exerted different antioxidant and anti-inflammatory effects depending on the plant considered. As cells were treated with the same dose of plant polyphenols (25 μM GAE), the different pattern of the regulation of ROS and cytokine production, SOD and NF-κB gene expression may result from the activity of some specific compounds present in the plants. For illustration, *D. apetalum* plant extract identified as the most bioactive extract contained procyanidins which were not detected in other plants. Moreover, a synergistic action of polyphenols depending on the plant extract considered cannot be excluded. This is in accordance with our previous work showing that antioxidant and anti-inflammatory properties of polyphenols may depend on their chemical nature, dose and ability to target cells according to their bio-accessibility extent [23].

In this study, we were interested in the effect of plant polyphenols on the production of three major pro-inflammatory cytokines, namely TNFα, IL-6 and MCP-1. However, TNFα was not detectable in 3T3-L1 preadipocytes. This agrees with previous data from authors who detected TNFα mRNA in 3T3-L1 preadipocytes, but were unable to measure any secreted TNFα [57]. A similar observation was reported by Fain et al., who found significant amounts of TNFα secreted by stroma vascular cells, with little or no detectable TNFα secreted by adipocytes obtained from human adipose explants [58]. The hypothesis suggested by the authors is that TNFα secretion by adipocytes would depend on signalling events from their *in vivo* environment, where they are exposed to macrophage-derived TNFα [57]. Collectively, our results led to suggest that the molecular mechanisms involved in the inflammatory state of adipose tissue during obesity could constitute relevant candidates for therapeutic agents such as antioxidant polyphenols. By demonstrating that polyphenol rich-extracts from medicinal plants increased cellular antioxidant defence system and down-regulated the release of pro-inflammatory molecules from preadipose cells, our data highlight their ability to interfere in the cross-talk between adipose cells and major immuno-competent cells like macrophages. This may result in an attenuation of the deleterious inflammatory process that occurs during obesity and related disorders such as type 2 diabetes. In order to assess the beneficial properties of polyphenols derived from medicinal plants tested here, it will be important to evaluate their effects in animal models, then in obese subjects. Such studies will also help to precise all systemic and tissular benefits, taking into account the bioavailability extent of polyphenols which constitutes a key factor governing their ability to efficiently target cells in *in vivo* condition.

## Conclusion

This work demonstrated that the medicinal plants *A. borbonica*, *D. apetalum* and *G. mauritiana* exhibited high levels of antioxidant polyphenols ranging from 1-7% GAE (w/w), among which flavonoids and two caffeic derivatives were identified, namely chlorogenic and ferulic acids. Polyphenol-rich extracts from these plants did not exert a cytotoxic effect on preadipocytes, but protected them against the anti-proliferative action of H_2_O_2_. They also down-regulated ROS production and the secretion of IL-6 and MCP-1 pro-inflammatory molecules from preadipocytes exposed to H_2_O_2_, TNFα or LPS mediators. Moreover, polyphenol-rich plant extracts increased SOD antioxidant enzyme gene expression and reduced mRNA levels of NF-κB pro-inflammatory transcription factor. Finally, our results raise the possibility that antioxidant polyphenols derived from medicinal plants tested could contribute to improve oxidative stress and inflammation within adipose tissue during obesity.

## Abbreviations

AAPH: 2,2′-Azobis [2-methyl-propionamidin] dihydrochloride
CE: Catechin equivalent
DCFH-DA: 2′,7′-Dichlorofluorescein-diacetate
DPPH: 2,2-Diphenyl-1-picrylhydrazyl
GAE: Gallic acid equivalent
GAPDH: Glyceraldehyde-3-phosphate dehydrogenase
UPLC-ESI-MS: Ultra-high performance liquid chromatography coupled to diode array detection and elestrospray ionization-mass spectrometry
IκB: Inhibitor-κB
IL-6: Interleukin-6
LDH: Lactate dehydrogenase
LPS: Lipopolysaccharide
MCP-1: Monocyte chemoattractant protein-1
MIP-1α: Macrophage inflammatory protein-1α
MTT: 3-(4,5-Dimethyl-thiazol-2-yl)-2,5-diphenyltetrazolium bromide
NF-κB: Nuclear factor-κB
ORAC: Oxygen radical absorbance capacity
ROS: Reactive oxygen species
RT-QPCR: Reverse transcription-quantitative polymerase chain reaction
SOD: Superoxide dismutase
TNFα: Tumor necrosis factor α
